# Cancer photo-immunotherapy: from bench to bedside

**DOI:** 10.7150/thno.53056

**Published:** 2021-01-01

**Authors:** Miao Wang, Jie Rao, Meng Wang, Xiaosong Li, Kaili Liu, Mark F. Naylor, Robert E. Nordquist, Wei R. Chen, Feifan Zhou

**Affiliations:** 1 School of Biomedical Engineering, Hainan University, Haikou, 570228, China; 2 College of Physics and Optoelectronic Engineering, Shenzhen University, Shenzhen, 518060, China; 3 Department of Oncology, the First Affiliated Hospital of Chinese PLA General Hospital, Beijing 100048, China; 4 Stephenson School of Biomedical Engineering, The University of Oklahoma, Norman, OK 73019, USA; 5 Baylor Scott & White Healthcare, Waco, Texas 76712, USA; 6 Immunophotonics, Inc., 4320 Forest Park Ave., #303 (BAL), St. Louis, MO 63108, USA

**Keywords:** Cancer, phototherapy, immunotherapy, photo-immunotherapy, combination therapy

## Abstract

Targeted therapy and immunotherapy in combination is considered the ideal strategy for treating metastatic cancer, as it can eliminate the primary tumors and induce host immunity to control distant metastases. Phototherapy, a promising targeted therapy, eradicates primary tumors using an appropriate dosage of focal light irradiation, while initiating antitumor immune responses through induced immunogenic tumor cell death. Recently, phototherapy has been employed to improve the efficacy of immunotherapies such as chimeric antigen receptor T-cell therapy and immune checkpoint inhibitors. Phototherapy and immunoadjuvant therapy have been used in combination clinically, wherein the induced immunogenic cell death and enhanced antigen presentation synergy, inducing a systemic antitumor immune response to control residual tumor cells at the treatment site and distant metastases. This review summarizes studies on photo-immunotherapy, the combination of phototherapy and immunotherapy, especially focusing on the development and progress of this unique combination from a benchtop project to a promising clinical therapy for metastatic cancer.

## Introduction

With developments in diagnosis and treatment techniques, tumor control at the early stages of cancer has been improved. However, late-stage cancers, especially those with metastasis, are still a major cause of treatment failure and death for patients with cancer 1. Cancer immunotherapy can activate and/or enhance the immune system to track and destroy tumor cells. Therefore, many immunotherapies have been used in clinical studies for treatment of metastatic cancer, including monoclonal antibody (mAb) therapy, cytokine therapy, vaccination, checkpoint inhibition, and chimeric antigen receptor (CAR) T-cell therapy 2, 3. In particular, the discoveries of dendritic cell (DC)-based vaccines and checkpoint inhibitors were recognized with Nobel Prizes in 2011 and 2018. Although cancer immunotherapies have achieved promising results in preclinical and clinical studies, control of metastases remains a big challenge 2, 3. In particular, systemic immunity is required to fight against metastases and prevent recurrence 4.

Cancer vaccines are designed to treat existing cancers by enhancing the natural defensive ability of the human body. They are usually made from substances taken from tumor cells or immune cells sensitized with tumor cells 5. The DC vaccine Provenge (sipuleucel-T) was the first active immunotherapy drug approved by the US FDA in 2011 for treatment of patients with prostate cancer 6. In a preclinical trial, neoantigen vaccines that minimize potential induction of central and peripheral tolerance, as well as reduce the risk of autoimmunity, were developed as personalized tumor vaccines for treatment of “cold tumors” 7, 8. However, the preparation of effective vaccines still faces challenges, particularly in the identification of immunogenic neoepitopes on different cancer cells.

A novel strategy for in situ cancer vaccination uses the patient's own tumor antigens that are produced by a local treatment such as phototherapy or radiotherapy. Phototherapy provides an elegant solution for ablating primary tumors due to its high specificity in light delivery, low level of trauma, and effectiveness in destroying target tumors 9. Because of the deep penetrability of near-infrared (NIR) light in biological tissues, NIR light can be used for phototherapy with corresponding *in situ* administered or natural absorbance agents 10. These photoagents convert absorbed light energy into heat for photothermal effects, as in photothermal therapy (PTT), or into reactive oxygen species (ROS) for photochemical effects, as in photodynamic therapy (PDT). A strategy called photoimmunotherapy (PIT) uses an NIR-absorbing photoagent conjugated to a mAb to target and destroy tumor cells under light irradiation. Phototherapies with appropriate photoagents and light doses have been found to induce immunogenic cell death (ICD) in target tumors with the release of tumor-associated antigens (TAAs) and damaged-associated molecular patterns (DAMPs), which may trigger a T helper 1 (Th1)-biased immune response 11-13. Additionally, PDT may cause necrosis and apoptosis in target cells and surrounding non-target cells, inducing an inflammatory response 14. Therefore, phototherapy provides sources of tumor antigens and DAMPs locally, creating a potential for generating in situ autologous tumor vaccines to prevent tumor progression and metastasis.

Photoagents should possess strong optical absorption at a therapeutic wavelength, high photothermal/photochemical conversion efficiency, and good biocompatibility. Many photosensitizers have been used in the clinic for PDT including porphyrins, indocyanine green, methylene blue, and Rose Bengal. However, a limited number of photothermal agents have been used in the clinic for PTT 15. Nanoparticles composed of metals, polymers, carbon, and lipids are considered ideal photothermal candidates due to their strong optical absorption and easily modulated structures 16, 17. Some nanoparticles have been developed for imaging-guided phototherapy, such as MoSe_2_/Bi_2_Se_3_ for high-contrast computed tomography (CT) imaging-guided PTT 18, and a biocompatible titanium nitride (TiN) nanoplatform for NIR-II laser-excited photoacoustic (PA) imaging-guided PTT 19, 20. Yang et al. synthesized a gadolinium ion-loaded thermally sensitive polymer nanoplatform for PA, magnetic resonance (MR), and positron emission tomography (PET) multimodal imaging-guided chemo-photothermal combination therapy 21. AuroShells are tiny silica spheres with a thin outer shell of gold that were developed for treatment of patients with prostate cancer. A recent feasibility study revealed that 13 of 15 prostate cancer patients evidenced no detectable signs of cancer a year after PTT with AuroShells 22. As the first clinical study of a nanoparticle-based PTT, this study showed great potential for further clinical applications.

Targeted approaches usually aim to inhibit tumor growth directly, whereas immunotherapies attempt to relieve immunoregulatory suppression or stimulate host immunity to achieve long-lived tumor control 23. Therefore, a combination of targeted therapy and immunotherapy is the ideal strategy to eliminate primary tumors while triggering systemic immunity to control residual tumors and distant metastases. Based on their synergistic thermal-immuno effects, combinations of photothermal agents and immunoadjuvants (e.g., LPS, CpG, R848) or cytokines (e.g., GM-CSF, G-CSF) as endogenous vaccinations have been developed in recent years 24-27. In addition, the introduction of checkpoint inhibitors (e.g., antibodies against PD‐L1 (programmed cell death-ligand 1), antibodies against CTLA-4 (cytotoxic T lymphocyte-associated antigen-4), small molecule IDO inhibitors (indoleamine 2,3-dioxygenase)) after phototherapy has been shown to markedly improve treatment efficacy by blocking the immunosuppressive receptors on the cell surface, thereby restoring the cytotoxic function of tumor-specific T-cells 28, 29.

**Figure 1:**
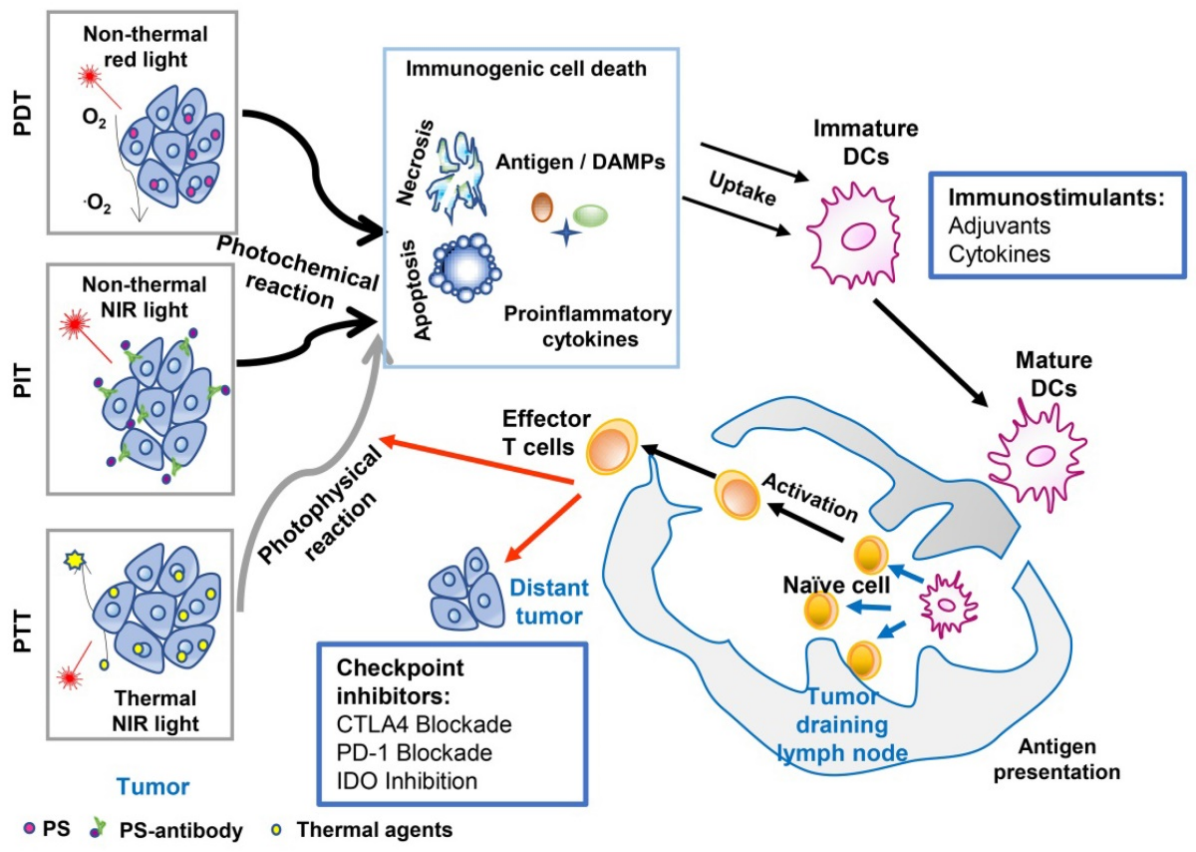
An overview of cancer treatment using the combination of phototherapy and immunotherapy. Agents absorbed energy from light to kill tumor cells, by photochemical reaction in the case of PDT (Photodynamic therapy) and PIT (Photoimmunotherapy), or photophysical reaction in the case of PTT (Photothermal therapy). Induced tumor cell death with the release of antigens, DAMPs, and proinflammatory cytokines, can provide *in situ* autologous cancer vaccines. Immunoadjuvants or cytokines can enhance the antigen capture and presentation by APCs, which will amplify the subsequent systemic immune response, resisting the residual tumor cells in the primary sites while allowing the host to establish a long-term defense against homologous cancer. Checkpoint inhibitors (antibodies against PD‐L1, antibodies against CTLA-4, or small molecule IDO inhibitors) can further improve the treatment efficacy by blocking the immunosuppressive receptors on the cell surface, restoring the cytotoxic function of tumor-specific T-cells.

The combination strategy of phototherapy and immunotherapy (photo-immunotherapy) has been found to achieve synergistic effects in the treatment of metastatic cancer, with an enhanced systemic immunostimulatory response (Figure 1) 30, 31. Phototherapy provides the first line of defense against the tumor, whether it is the original or recurrent tumor, either the same or mutated. More importantly, phototherapy releases antigens, DAMPs, and other tumor components, providing a source for activating immune system. Therefore, photo-immunotherapy can overcome the challenges of tumor heterogeneity, tumor mutation, tumor immune editing, and escape. In particular, phototherapy combined with immunoadjuvant has been utilized to treat patients with advanced cancer 32-35. It is expected that photo-immunotherapy will experience continuous progress both in basic and clinical research. This review summarizes the development of cancer treatment using this unique combination and introduces its progress in clinical studies.

## Photothermal immunotherapy

Photothermal immunotherapy, a novel concept combining PTT with immunotherapy, can lead to a synergistic thermal-immune effect and trigger a specific antitumor immunity 36. PTT can directly target and destroy the primary tumor by increasing the temperature of the tumor tissue, a strategy that has been used for ablation of glioma, hepatocellular carcinoma, lung cancer et al. 37, 38. PTT induces a temperature gradient within the tumor tissue that leads to various biological responses from cell stress to cell death 39, 40. An appropriate temperature gradient can cause a high level of cell death with antigen exposure and DAMPs release, including the exposure/release of HSPs (heat shock proteins), ATP (adenosine triphosphate), and HMGB1 (high-mobility group box 1) 39, 40. This type of cell death is called immunogenic cell death (ICD) and it provides potential for enhancing APCs activation and antigen presentation. However, high temperature would cause tumor eradication with denatured proteins, and simultaneously attack surrounding normal tissues and inhibit the host immunoreaction 41. PTT inducing ICD has been developed in combination with immunotherapies such as CAR T-cell therapy, checkpoint inhibitors, and immunoadjuvants to enhance control of both primary tumors and metastases. With nearly two decades of research, photothermal immunotherapy has been developed from a concept to a benchtop project and is finally a promising clinical therapy for metastatic cancer 32, 42.

### Preclinical studies of photothermal immunotherapy

#### Combination of PTT with immunoadjuvant

**Figure 2:**
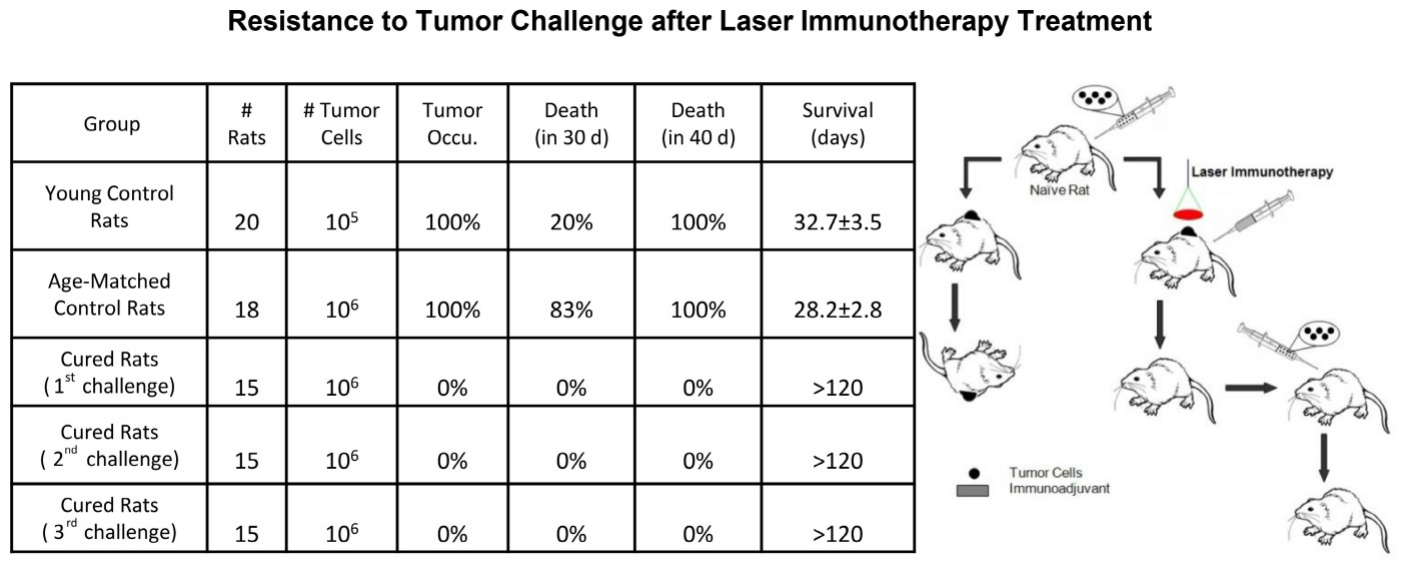
Tumor rechallenge of rats cured by LIT. Left table is the resistance to tumor challenge after laser immunotherapy treatment. The table is adapted with permission from 45, copyright 2001 American Association for Cancer Research. Right figure is the schematic of anti-tumor immunity induced by laser immunotherapy in tumor rechallenge. The figure is adapted with permission from 36, copyright 2015 Elsevier Ireland Ltd.

The combination of target PTT and *in situ* administration of an immunoadjuvant, formally referred to as laser immunotherapy (LIT), was first proposed in 1997 43. LIT was found to induce a host antitumor immunity that eliminated primary residual tumor cells and distant metastases 44. In this early study of LIT, non-invasive PTT was performed using an 805 nm diode laser and the photoagent indocyanine green (ICG). N-dihydrogalactochitosan (GC), a functionalized soluble chitosan, was used as the immunoadjuvant. The therapeutic effects of LIT were first studied in a metastatic rat mammary tumor model, DMBA-4. LIT treatment (laser-ICG-GC) resulted in a 33% tumor-free survival rate versus 0% survival in the control group 44. It is noteworthy that some of the surviving rats had metastases at an early stage that gradually subsided after LIT. In addition, all the cured rats showed complete resistance to 10-fold tumor cell rechallenge (Figure 2) 45.

To further confirm the immune-stimulating ability of GC, the effects of four different immunostimulants combined with laser-ICG were evaluated in the DMBA-4 model: 1% GC aqueous solution, 50% complete Freund's (CF) adjuvant, 50% incomplete Freund's (IF) adjuvant, and *Corynebacterium parvum* (CP) 46, 47. Compared with the control group, all immunostimulants (CF, IF, and CP) significantly improved the survival rate of animals, increasing the cure rate from 0% to 18%, 7% and 9%, respectively. In contrast, the survival rate of rats induced by GC was 29%.

**Figure 3:**
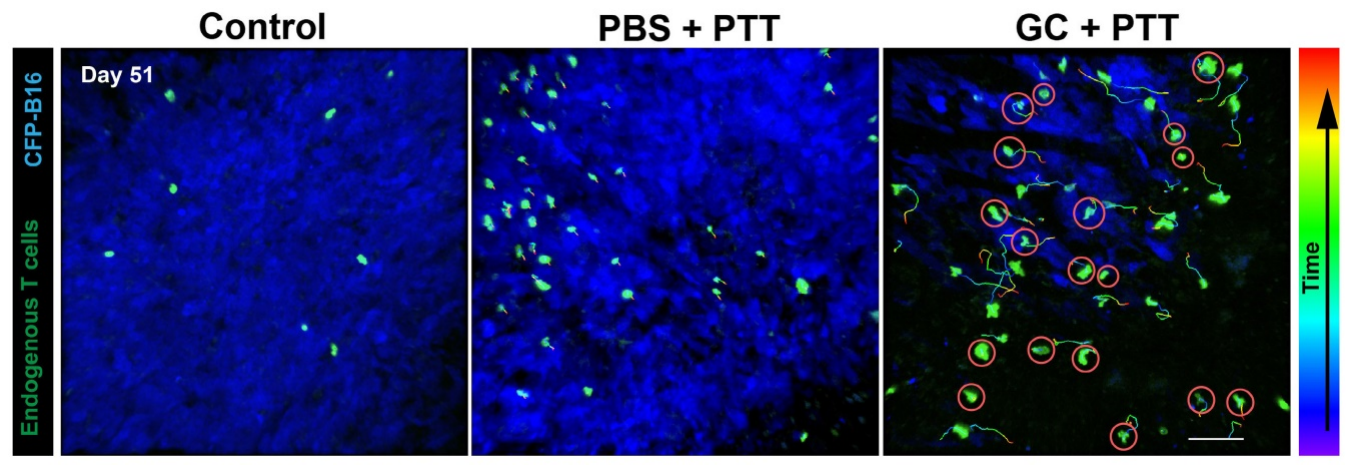
LIT (PTT+GC) induced tumor-specific immune memory. In vivo time-lapse images showing migration of endogenous GFP^+^ TILs in the CFP-B16 tumor area of CXCR6-GFP mice treated by LIT and rechallenged with CFP-B16. Scale bar: 70 μm. The figure is adapted with permission from 50, copyright 2020 Ivyspring International Publisher.

Recently, non-invasive interstitial PTT without administered photoagents has been achieved by directly delivering laser energy into the tumor tissue with an optical fiber. GC combined with PTT using a 980 nm laser resulted in a cure rate of 70% in mice bearing subcutaneous EMT6 mammary tumors and resulted in a cure rate of 75% in mice bearing subcutaneous Panc02-H7 pancreatic tumors 48, 49. In particular, LIT treatment of orthotopic pancreatic cancer in mice destroyed primary tumors, slowed metastases, and prolonged survival 49. During this treatment, the process of the immune response was investigated. Exposed/released DAMPs were detected in primary tumor treated by PTT, which initiated infiltration of APCs and a Th1 immunization. The combined regimen of GC-amplified Th1 immunization increased the quantity of cytotoxic T lymphocytes (CTLs) to control residual tumor cells. Simultaneously, the collaborative strategy also led to tumor-specific immune memory, inhibiting tumor recurrence and producing memory T-cells 49. In another study, intravital imaging of CXCR6-GFP mice bearing CFP-B16 melanoma showed that infiltration of tumor-infiltrating lymphocytes (TILs) was increased with highly active motility at recurrent sites in mice treated with PTT+GC (Figure 3) 50.

Due to their strong light absorption and carrier capabilities, nanoparticles have been employed for photothermal immunotherapy. Zhou et al. developed single-walled carbon nanotubes (SWNTs) to deliver GC intracellularly. With the synergized photothermal and immunostimulation effects, CTL response was amplified to control remote EMT6 tumor and memory immunity was triggered to resist tumor rechallenge 48. Guo et al. constructed chitosan-coated hollow CuS nanoparticles (HCuSNPs) with oligodeoxynucleotides (ODNs) containing cytosine-guanine (CpG) motifs as immunoadjuvants for PTT. In an EMT6 mouse model, synergized photo-immune effects destroyed the tumor and controlled metastasis 51. In another study, the immunoadjuvant Resiquimod R848 was loaded into polydopamine nanoparticles with co-loaded carbon dots. PTT with this nanoconstruct eliminated primary 4T1 breast tumors and triggered infiltration of CTLs into distant tumors 27. Many novel synergized nanosystems have been designed using similar strategies for cancer therapy 52.

#### Combination of PTT with checkpoint inhibitors

Immunological checkpoint inhibitors break tumor immune tolerance by blocking immunosuppressive receptors on the cell surface, which restores the cytotoxic function of tumor-specific T-cells 53. Currently, the main immunological checkpoint inhibitors include CTLA-4 mAb and PD-1/PD-L1 mAb. CTLA-4 is a cell surface transmembrane receptor that is induced by T-cell receptor binding and acts as a negative regulator of antigen-specific T-cell activation. Binding of anti-CTLA-4 antibodies to CTLA-4 receptors on Treg cells induces evasion of this immune checkpoint to reduce T-cell-activated immunosuppression. Yervoy (Ipilimumab) was the first immunological checkpoint inhibitor approved by the FDA in 2011 for treatment of metastatic melanoma. Yervoy acts by blocking the activity of the “brake” protein CTLA-4 on T-cells and restoring the immune system's ability to fight cancer 54. PD-L1, a ligand of PD-1 expressed in many types of tumors, has been shown to inhibit T-cell receptor-mediated activation of positive signals. Two PD‐1 pathway inhibitors, Pembrolizumab and Nivolumab (biological mAbs), have been approved by the FDA 55. However, infiltrating T-cells are the prerequisite for effective anti-CTLA-4/PD-1 therapy. In addition, not all tumors express ligands binding to CTLA-4 and PD-1, and anti-CTLA-4 and anti-PD-1 mAbs are relatively less effective at ablating solid primary tumors.

Based on the systemic antitumor immune response induced by PTT or LIT, checkpoint inhibitors have been included to magnify the effects of CTLs. Li et al. reported that combining laser-SWNT-GC treatment with anti-CTLA-4 promoted synergistic immunomodulatory effects and further prolonged the survival time of 4T1 tumor-bearing mice 56. Yang et al. reported the construction of a Au@Pt-LMDP nanoplatform with a designed peptide antagonist of PD-L1 (LyP-1-PLGVRG-DPPA-1, LMDP) for cancer photothermal immunotherapy 57. Zhang et al. synthesized IR820-1MT nanocomposites as photothermal agents for the combination of PTT and IDO inhibitor 29. Qian et al. linked ICG to imiquimod, an immunoadjuvant, by poly lactic-co-glycolic acid, providing an *in situ* vaccination for antitumor immune response, which was further enhanced by anti-CTLA-4, a checkpoint inhibitor, to inhibit metastasis 58.

#### Combination of PTT with other strategies

Currently, CAR T-cell therapy has been combined with PTT to treat melanoma 59. T-cells with CAR have been used to treat a number of patients with lymphoma and leukemia. However, CAR T-cell therapy has had limited success in the treatment of solid tumors due to the protective microenvironment. A study reported that the combination of photothermal ablation and CAR T-cells inhibited the growth of melanoma tumors in mice for up to 20 days. After receiving the combination therapy, 33% of mice remained tumor free after 20 days 59. Multimodal therapies have also been developed, such as the synergistic combination of PTT, immunotherapy, and doxorubicin (Dox) chemotherapy in the light-responsive biodegradable nanoparticle Dox/CAT-PDA@M. This nanoparticle delivery strategy resulted in higher accumulation (15.3%) versus the retention effect of conventional nanodrugs (7.5%) and complete tumor elimination *in vivo* 60.

### Clinical studies of photothermal immunotherapy

The encouraging results from animal studies have prompted clinical applications of photothermal immunotherapy. In particular, LIT has been used to treat patients with late-stage cancer who have failed other feasible treatment modalities. Preliminary clinical studies have shown that photothermal immunotherapy can reduce primary tumors, control untreated metastases, and prolong survival of patients. However, these studies were nonrandomized, investigator-driven, and included a limited number of patients 32-34, 42.

#### Photothermal immunotherapy for advanced breast cancer

The treatment of breast cancer has been significantly improved due to developments in technologies for early diagnosis and targeted therapies 61. However, advanced breast cancer is still the leading cause of death in women, especially in underdeveloped countries, due to limited access to either earlier diagnosis or advanced therapies. LIT was used in an investigator-driven preliminary clinical study to treat patients with breast cancer in South America using ICG as the photoagent and GC as the immunoadjuvant. The treatment was performed every 4 weeks for a total of 4 times as one cycle. An additional treatment cycle was carried out if viable targeted tumor tissue remained.

In a study conducted in Lima, Peru, ten patients with breast cancer (stage III or stage IV) were enrolled in a photothermal immunotherapy clinical trial 32. All the patients had failed conventional therapies and had a life expectancy of 3-6 months at the time of enrollment. The patients only experienced minor side effects in the area of treatment, without any grade 3 or 4 side effects. In eight patients available for evaluation 3 years after treatment, there were 2 patients with complete response (CR) and 2 patients with partial response (PR). The overall clinical benefit rate (CR+PR) was 50% (unpublished data). Significant reductions of metastases in lymph nodes, lungs, and livers were observed in 4 patients. All lung metastases disappeared in one complete responder. The overall 3-year survival of evaluable patients was significant, giving the fact that these patients had a life expectancy of 3-6 months prior to the study.

#### Photothermal immunotherapy for melanoma

Although melanoma accounts for only 4% of skin cancer cases, it leads to 79% of all skin cancer deaths 62. The prognosis of metastatic melanoma is very poor, with only a 5% long-term survival rate. Photothermal immunotherapy of melanoma using imiquimod, an FDA-approved toll-like receptor agonist, as the immunoadjuvant was previously referred to as in situ photoimmunotherapy (ISPI) 33, 34. Topical application of imiquimod was found to be effective on advanced melanoma, even when used as a monotherapy 63. Its immunostimulatory effect makes imiquimod a reasonable candidate for photothermal immunotherapy.

In 2006, Naylor et al. reported initial results from the treatment of two patients using ISPI 34. Patient 1 had primary tumors and regional metastases on the left arm with pulmonary metastases (AJCC stage IV). Patient 2 had primary melanoma on the head and neck with regional metastases (AJCC Ⅱ IC stage). The second patient failed multiple surgical resections and numerous cycles of high-dose radiotherapy. Both patients received ISPI and eventually cleared all detectable tumors. Both patients had no clinically detectable tumor (including pulmonary metastasis) for more than 60 months. In 2010, Murda et al. reported the use of ISPI for the treatment of a patient with acral lentiginous melanoma with metastases in an inguinal sentinel lymph node and on the left leg 64. Two subcutaneous nodules were treated on the dorsal side of the left foot. The treatment was well tolerated by the patient with no serious adverse events and only a few minor side effects such as local wound ulcers and local pain. Five weeks after ISPI, one non-targeted adjacent tumor showed clinical remission, while another non-targeted lesion remained unchanged. The recurrence rate was decreased, with only 3 new nodules compared to 12 recurrences prior to ISPI treatment. When a new or recurrent nodule appeared, the patient continued to receive further ISPI to support the initial goal of limb salvage. There was no detectable visceral metastasis 18 months after the initiation of ISPI. These three cases showed that ISPI could eliminate local tumors and induce beneficial systemic reactions. Its side effects were more favorable than other methods in the treatment of advanced melanoma.

Li et al. reported results of a preliminary clinical study to determine the safety and efficacy of ISPI on metastatic melanoma patients 33. Eleven patients with advanced melanoma (stage III or stage IV) were enrolled in the clinical trial. The most serious side effects usually occurred in the first treatment cycle. Approximately 20% of patients felt obvious pain during laser treatment and usually responded to pre-oral administration of anesthesia, although one patient needed conscious sedation. There were no level 4 adverse events in this study. In ten patients available for evaluation after treatment, 6 showed CR, and 2 showed PR. All lesions in the treatment area responded to local laser irradiation, and 8 cases achieved complete local response (CLR). Notably, 4 cases also achieved CLR of lesions in the nontreatment site, implying that the combination modality may induce an abscopal effect to treat metastatic melanoma. A summary of this study is given in Table 1. The overall 12-month survival rate reached 70%, and the survival distribution was never less than 50%. Another important outcome of this study is that the surviving patients continued to maintain a good quality of life after ISPI treatment.

ISPI therapy for advanced melanoma patients stimulates a long-term adoptive immune response against residual primary and metastatic cancer tissues 33, 34, which may increase the quantity of anti-tumor T-cells, setting the stage for enhanced effects with a checkpoint inhibitor such as ipilimumab. Therefore, the combination of ISPI and ipilimumab was developed to treat patients with advanced melanoma 35. In one particular patient, all melanoma nodules on the head and neck treated by ISPI achieved CLR. However, there were no changes in pulmonary metastases. After the implementation of ipilimumab, all the pulmonary metastases were decreased and finally disappeared. This patient remained tumor free and healthy seven years after treatment. This patient's response supports the hypothesis that ISPI increases the number and quality of T-cells in the tumor microenvironment, making the treatment more effective in combination with ipilimumab and other checkpoint inhibitors.

Dinitrophenyl (DNP), a type of hapten, can lead to contact-delayed hypersensitivity (DTH) due to its strong antigenicity and effective absorption in normal skin. DTH is a T-cell-mediated immune response, which is induced by an allergen, with the function of human cellular immunity evaluation 65. DNP hapten combined with PTT to treat patients with malignant melanoma significantly increased interferon‑γ production by CD8^+^ and CD4^+^ T-cells and reduced secretion of IL‑10, TGF‑β1, and TGF‑β2, resulting in significantly longer overall survival and disease‑free survival 66.

While photothermal immunotherapy has been used in preliminary clinical studies for breast cancer and melanoma, it has limitations. In these applications, non-invasive light delivery was employed, which limits treatment to surface tumors. Interstitial photothermal immunotherapy has been experimented within pre-clinical studies and in limited clinical studies, which can extend the use of photothermal immunotherapy to treat deep-seated tumors or tumors in internal organs. Imaging-guided photothermal immunotherapy and nanotechnology-assisted photothermal immunotherapy will further broaden the applications of photothermal immunotherapy.

## Photodynamic immunotherapy

Photodynamic therapy (PDT) is a targeted treatment based on local activation of a photosensitizer (PS) by light at a corresponding wavelength, which leads to ROS generation and induced tumor cell death, which has been used to treat cancer in the clinic for more than 40 years 15. To achieve higher precision and deeper tissue penetration, interstitial photodynamic therapy (iPDT) has been used to destroy large, deep-seated tumors by directly transferring light into solid tumors through fiber optics inserted under imaging guidance 67. While PDT has become a mature cancer therapy, it has undesirable side effects such as prolonged phototoxicity due to systemic administration of photosensitizers. In addition, new PSs have been developed that are capable of absorbing two photons of long-wavelength light 68. At present, due to improvements in second- and third-generation photosensitizers, such as biocompatible BiOI/BiOIO_3_ heterostructure nanocomposites (BB NCs), and nanoscale metal-organic frameworks (nMOFs), an increasing number of trials are underway to improve the feasibility and efficacy of PDT 16, 69-71. PDT with appropriate photosensitizers and light doses can enhance antitumor immune responses by releasing antigens and danger signals, supporting the combination of PDT with immunotherapy 72. PDT-based tumor vaccines have been developed and enhanced by combination with immunostimulants and immunological checkpoint inhibitors 73.

### Combination of PDT with mAbs

NIR-photoimmunotherapy (NIR-PIT) is a cell-selective cancer therapy that uses cancer-targeting antibodies (mAbs) conjugated to a nontoxic NIR-absorbing PS that can be activated at the tumor site with light of a corresponding wavelength. The antibody-PS conjugate binds to cancer cells that overexpress the targeted cancer-associated antigens. NIR light activates photochemical reactions that make the hydrophilic antibody-PS become hydrophobic, leading to the formation and accumulation of antibody-antigen complexes on the membrane with physical stress, resulting in increased transmembrane water flow, and ultimately causing cell rupture and necrosis 14, 74. Moreover, PIT-mediated rapid cell death with associated release of tumor-associated antigens and membrane damage signals, induces maturation of local DCs and promotes tumor-specific naïve T-cell activation and proliferation 75.

#### Preclinical study of PIT

In 1983, the term PIT was used for cancer treatment involving chemical phototoxicity and antibody targeting 76. Chemically coupled hematoporphyrin and a mAb of DBA/2J myoma M-1 was found to suppress M-1 tumor growth following exposure to incandescent light. A number of photosensitizers (e.g., mTHPC, pheophorbide a (PPa), chlorin e6 (Ce6)) and various mAbs have been used for PIT 77-80. However, the success was very limited to use in vivo, because these conjugates were trapped and catabolized in the liver due to the hydrophobicity of photosensitizers soon after intravenous injection. In 2011, an EGFR mAb conjugated to a phthalocyanine dye (IRDye 700DX), initially discovered by Kobayashi and coworkers, was found to induce the death of EGFR-expressing cells immediately upon irradiation 81. The photosensitizer benzoporphyrin derivative (BPD) was conjugated to Cetuximab, an FDA-approved anti-EGFR mAb, for selective treatment and quantitative, longitudinal imaging of micrometastases in vivo in an advanced ovarian cancer model 82.

Since the discovery of PIT, various antibodies have been conjugated to IRDye 700DX to target different cells 83. For example, the HER2-specific antibody trastuzumab was synthesized with IRDye 700DX and administered to a non-small-cell lung carcinoma mouse model and a HER2-positive model of disseminated peritoneal ovarian cancer. PIT led to significant reductions in tumor volume in both the flank model and the pleural dissemination model 84-89. Similarly, the delta-like protein 3 (DLL3) mAb rovalpituzumab was conjugated to IRDye 700DX to form rova-IR700, which decreased tumor burden markedly by PIT 90. An anti-podoplanin antibody, NZ-1, was also synthesized with IRDye 700DX to treat a malignant pleural mesothelioma (MPM) model 74. Further, anti-PSMA-IR700 was developed by conjugating IRDye 700DX to a full human IgG1 anti-PSMA mAb for the treatment of prostate cancer. PIT with this construct inhibited tumor growth and prolonged survival 91.

Although PIT has been shown to destroy target tumor cells and induce ICD, in most cases, it failed to induce durable antitumor responses against homologous tumor. Therefore, Kobayashi et al. combined CD44-targeting PIT with PD-1 blockade and evaluated its therapeutic effects in multiple syngeneic tumor models 92. Because PD-1 blockade enhances tumor antigen-specific T-cell response, this combination led to complete rejection of MC38 tumors and untreated distant tumors. In addition, CD25-targeted PIT was developed to selectively clear Tregs. This therapy activated CD8^+^ T-cells and natural killer cells and restored local antitumor immunity, resulting in regression of treated tumors and separate untreated tumors 93.

#### Clinical study of PIT

RM-1929 is a parenteral formulation that consists of a chemical conjugate of the dye IR700 with an FDA-approved EGFR receptor-targeting antibody. In July 2015, a Phase I clinical trial was initiated to evaluate the safety and antitumor activity of RM-1929 in patients with terminal head-and-neck cancer (NCT number: NCT02422979). Nine patients were enrolled in three cohorts for dose escalation studies. No dose-limiting toxicities were found. The objective response rate (ORR) was 75% (6/8), with 3 complete responders for the duration of 4-16 months. In 7/8 of patients, the tumor density was decreased, consistent with necrosis after treatment. The disease control rate (DCR) was 100% 94.

Thirty patients with recurrent head-and-neck squamous cell carcinoma (rHNSCC) were enrolled in a Phase 2 study in June 2016 to evaluate the safety and anticancer efficacy of repeated treatment with PIT (four times under the maximum feasible dose of RM-1929 with a fixed dose of red light). Thirteen patients (43.3%) had at least one serious adverse event (SAE). A total of 86% (19/22) of SAEs were not considered to be related to treatment, including three kinds of fatal SAEs. Three kinds of SAEs may have been related to treatment (i.e., site/oral pain, tumor hemorrhage, and airway obstruction). The ORR was 50% (15/30), with 16.7% (5/30) CR and 86.7% (26/30) DCR 95. For patients with locally recurrent head-and-neck squamous cell carcinoma who failed after at least two treatments, a Phase 3 randomized, double-arm, open-label trial of PIT started in May 2019. In addition, the NIR-PIT was approved in human clinical use by Japanese Agency, in September 2020.

### Combination of PDT with other immunotherapies

Various immunoadjuvants have been used in combination with PDT, such as schizophyllan (SPG), GC, Bacille Calmette-Guérin (BCG) and mycobacterium cell wall extract (MCWE) 80. In the treatment of line 1 lung adenocarcinoma in mice, a poorly immunogenic tumor model, 37% of the tumor-bearing mice were cured by the combination of 1.67% GC solution with noncurative PDT using m-tetra (hydroxyphenyl) chlorin (mTHPC) 47. Combinations of BCG or MCWE with PDT using different photosensitizers, including Photofrin (porfimer sodium), BPD, zinc phthalocyanine, mTHPC, mono-L-aspartyl-chlorin e6, and lutetium texaphyrin, all resulted in delayed tumor regrowth and an increased cure rate in experimental animals 80, 96.

Synthetic long peptides containing epitopes from tumor antigens were combined with Bremachlorin-based PDT for the treatment of TC-1 and RMA aggressive mouse tumor models. The treatment cured one-third of tumor-bearing mice and cured mice completely resisted tumor rechallenge 97. The checkpoint inhibitor nivolumab was combined with Redaporfin-mediated PDT for the treatment of a patient with head-and-neck cancer 98. The patient was diagnosed with SCC of the oral floor and had failed surgery, radiotherapy, and multiple lines of systemic treatment. PDT destroyed all visible tumors, while sequential use of nivolumab produced a sustained complete response.

Using strategies similar to those employed in nanoparticle-based photothermal immunotherapy, numerous nanosystems have been designed for photodynamic immunotherapy, including metal materials or organic nanoparticles as PSs combined with immunoadjuvants or checkpoint inhibitors. Lin et al. designed a novel up-conversion luminescence (UCL)-enhanced spindle-shaped nanocomposite for mitochondrial imaging that is coated with gold nanoparticles for PDT (SPS@Au), and is combined with anti-CTLA-4 mAb for photodynamic immunotherapy 99. Recently, multifunctional nanoparticles have been developed to synergize PDT, chemotherapy, and immunotherapy, such as a TME-triggered oxygen nanogenerator PTX/ICG-NVs@Au@CAT 100. In this nanoconstruct, H_2_O_2_ is catalyzed to oxygen by CAT to relieve tumor hypoxia, enhancing ROS generation by ICG-PDT, which potentiates the efficacy of immunotherapy.

### Clinical studies of photodynamic immunotherapy

Imiquimod has been combined with PDT for the treatment of patients with skin cancer. In one trial, 10 days after two sessions of PDT treatment, patients received imiquimod 5 times a week for 3 weeks, which resulted in clinical clearance and no recurrence at 15 months 101. Osiecka et al. reported a trial of 10 patients who underwent PDT treatment with 5-aminolevulinic acid (ALA) and 24 patients who underwent ALA-PDT combined with imiquimod. In the first group, 6 cases (60%) were completely cured and 4 cases (40%) had reduced lesions; in the second group, 18 cases (75%) had complete lesion erasure and 6 cases had reduced lesions 102. Requena et al. developed a trial of recurrent giant basal cell carcinoma of the face, showing excellent efficacy with methyl aminolevulinate (MAL)-based PDT plus imiquimod treatment 103. Another randomized, prospective, observer-blinded study of photodynamic vaccination in patients with non-melanoma skin cancer (NMSCs) was started in 2013 to determine the enhanced efficacy of PDT in combination with 5% imiquimod in 44 patients (EudraCT ID: 2013-000092-33). MAL‐PDT and imiquimod did not show improved efficacy in reducing growth of new recurrent NMSCs at any follow‐up period compared to MAL-PDT alone. Both therapies were safe and acceptable. Patients preferred MAL‐PDT based on the treatment strategy modalities, response rates, and future choice 104.

The combination of PDT with different immunological approaches provides possibilities to improve treatment efficiency. However, there are few clinical trials including immunotherapy as an adjunctive therapy for PDT. PDT-generated vaccine is a promising tool to induce tumor-specific immunity. With an understanding of the anti-tumor immune response triggered by PDT, combination therapies that can be used with PDT to target and potentiate the immune response will be further developed.

## Summary and outlook

Immunotherapy uses the host's immune system against cancer cells. Phototherapy is a promising modality that eradicates tumors through targeted light irradiation. The tumor treated by phototherapy serves as an *in situ* source of cancer antigens that come from the patients themselves, resulting in an *in situ* autologous whole-cell cancer vaccination. Therefore, the strategy of photo-immunotherapy is to destroy the tumor directly, induce a tumor-specific host immune response, and eliminate residual tumor cells and distant metastases. Clinical results show the potential of photo-immunotherapy for effective, local, and safe interventions for metastatic cancer.

However, phototherapy still has limitations in the treatment of human cancer, especially the limited tissue penetration of light, which restricts the non-invasive application of phototherapy for tumors in deep organs. Therefore, non-invasive phototherapy is suitable for superficial cancers such as melanoma, osteosarcoma, and squamous cell carcinoma. To circumvent these barriers, interstitial phototherapy was developed. Using interstitial fibers, phototherapy can treat deep-seated tumors and avoid damage to healthy tissue. Recently, MR-guided laser interstitial thermal therapy (LITT) has been used for treatment of brain tumors 71. Moreover, Hu et al. developed interventional PTT under CT imaging guidance and Bryan et al. developed interventional PDT, both using an orthotopic xenograft model of human pancreatic cancer 73, 74. Therefore, with the development of interventional and imaging techniques, imaging-guided phototherapy will be used widely for either epidermal tumors or deeper tumors, although further studies are needed for clinical applications.

For nanomaterial-based phototherapy, another challenge is the removal of nanoparticles from circulation by cells in the liver, spleen, and other parts of the reticuloendothelial system (RES), which may induce toxicity and reduce nanoparticle accumulation in tumor tissue. Therefore, intratumoral administration is commonly used to overcome RES uptake and low tumor targeting efficiency. Furthermore, PEG or zwitterionic polymers are often chosen to modify the surface of nanoparticles to avoid RES cells and increase accumulation in the tumor 105, 106. In addition, due to the differences and complexities of human tumor microenvironments compared with those of animal models, it is impossible to predict the clinical performance of photo-immunotherapies from *in vivo* results.

It has been recognized that phototherapy can initiate immune responses when treating tumors. It is further recognized that phototherapy alone usually cannot initiate and sustain curative anti-tumor immune responses. As introduced in this review, a combination of phototherapy and immunotherapy, particularly using immunostimulants, immune-targeting agents, and checkpoint inhibitors, can significantly advance cancer treatment. The effect of such combinations has resulted in promising clinical outcomes using PTT, PDT, and PIT with different immunotherapeutic agents, as shown in Sections 2 and 3.

In summary, with continued development of photo-immunotherapies, expanded preclinical and clinical studies, as well as further investigations of the mechanisms of photo-immunotherapy-induced antitumor immune responses, photo-immunotherapy will attract increasing attention. The potentials of photo-immunotherapy should be achieved for the treatment of patients with late-stage, metastatic cancers.

## Figures and Tables

**Figure 4:**
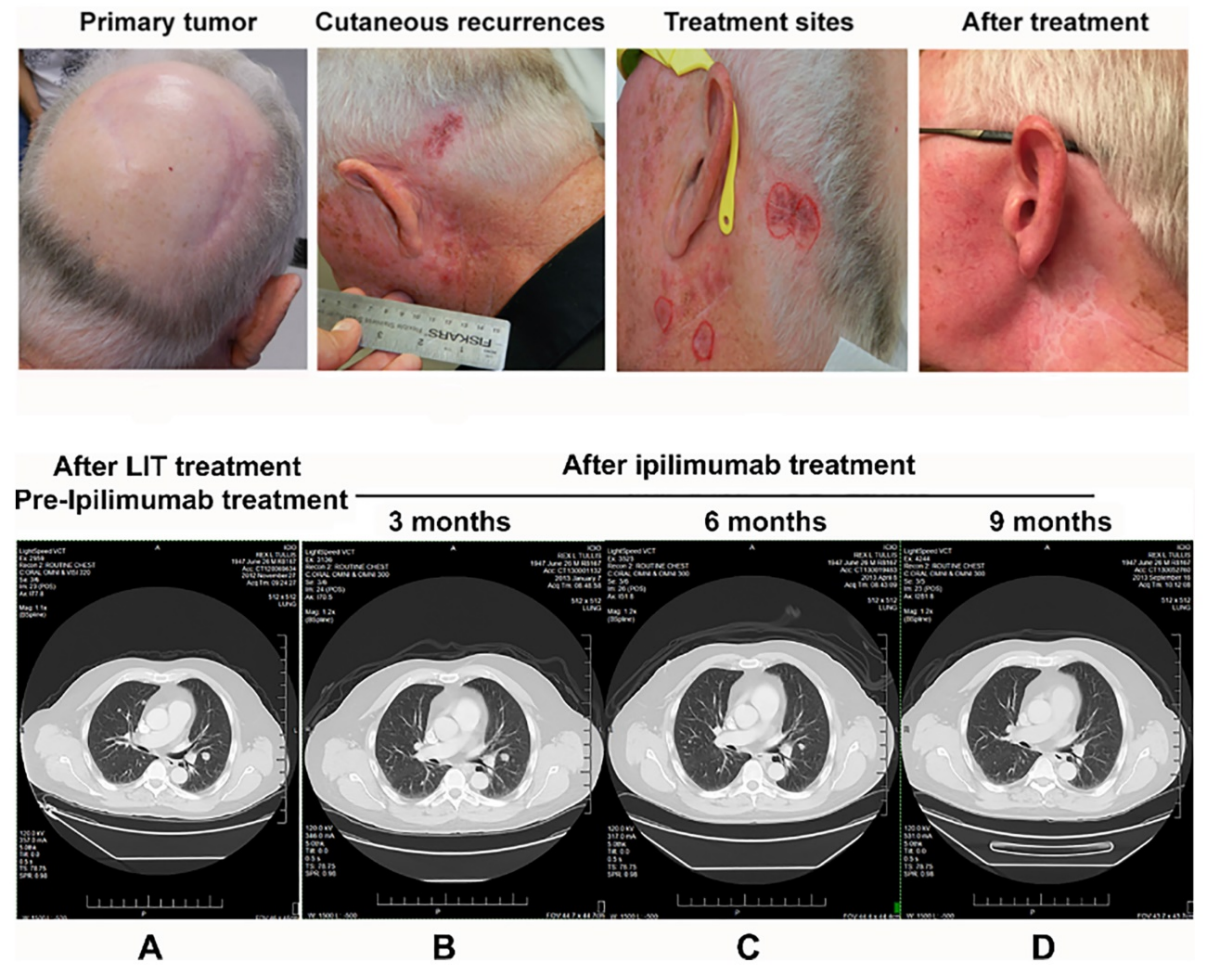
Photographs and CT images of a patient with stage IV melanoma during treatment. **Upper:** Photographs of the LIT treatment areas around the left ear. **A.** The primary tumor site with only surgical scars visible. **B.** The areas of biopsy-proven cutaneous recurrences around 2 surgical scars from the biopsy sites. Scalp hair has been trimmed away from the scalp recurrence site to make superficial laser therapy more effective. Reddish areas around the surgical scars represent melanoma deposits in the superficial dermis. **C.** Areas circled in red represent the 4 treatment sites selected for LIT. All other areas of regional involvement completely resolved during the 4-week treatment phase, which is typically seen with superficial LIT. **D.** The treatment area after LIT. Three months later, the LIT treatment completely cleared all cutaneous melanoma around the left ear. **Bottom:** CT images of the patient taken 3 months apart showing the same level in the thorax. **A**. The image was taken before ipilimumab treatment (2 months following LIT) and demonstrates the size and location of the pulmonary metastases. **B-C**. The images show shrinkage of the pulmonary metastases 3 months (**B**) and 6 months (**C**) after completion of the combination of LIT and ipilimumab. **D.** The image shows that pulmonary metastases were completely resolved 9 months after completion of the combination of LIT and ipilimumab. The figure is adapted with permission from 35, copyright 2017 Wiley‐VCH Verlag GmbH & Co. KGaA, Weinheim.

**Table 1:** Clinical characteristics of patients with melanoma and objective response

No.	Age	Sex	AJCC stage	Primary site	Initial metastatic sites*	Cycles of treatment	Treatment site response	Non-treatment site regional response	Best overall response	Time to response (months)	Response duration (months)	Overall survival (months)
**1**	64	F	IV M1b	Left forearm	Bilateral lower lobe lung	3	CLR	CLR	CR	1	12	66.4
**2**	67	M	IIIC	Left side of head	-	3	CLR	CLR	CR	4	7	64.2+
**3**	46	F	IV M1c	Back	perihilar region of lungs, liver, uterus	2	PLR	N/A	PR	1	2	8.5
**4**	63	F	IIIB N2c	Left leg	-	2	PLR	PLR	PR	3	4	44.9+
**5**	60	F	IV M1A	Right frontal scalp	Distal skin site	2	CLR	CLR	CR	2	4	6.6
**6**	87	M	IIIC N2c	Right foot (plantar)	-	6	PLR	SD	SD	1	0	15.5
**7**	71	M	IV M1c	Eye	Liver	1	CLR	N/A	PD	1	2	2.3
**8**	74	M	IIIC N3	Left lower back	-	1	CLR	N/A	CR	8	2	38.7+
**9**	84	M	IIIB N2c	Left foot	-	1	CLR	SD	CR	6	1	6.0**
**10**	85	M	IV M1c	Left arm	Anterior mediastinum	1	CLR	CLR	CR***	1	8	22.5+
**11**	69	M	IV M1c	Right forehead	Bone, lung	1	CLR	N/A	CR	1	6	20.6+

**Table 1.** All the treatment area lesions of enrolled patients responded to ISPI. Abbreviations: M, male; F, female; AJCC, American Joint Committee on Cancer; CLR, complete local response; PLR, partial local response; CR, complete response; PR, partial response; SD, stable disease; PD, progressive disease; N/A, not available or not applicable. *In addition to skin, present at the time of study entry. **This patient died of progression of myelodysplasia to leukemia (unrelated tumor death). ***The metastatic lesion was treated with cyberknife. Table 1 is adapted with permission from 33, copyright 2010 Landes Bioscience.

**Table 2:** Summary of studies utilizing phototherapy and immunotherapy combinatorial treatments

Phototherapy	Phototherapy agent	Immunotherapy	Immunotherapy agent	Cancer model	Therapeutic outcome	Ref.
**PTT**	**CuS**	Immunoadjuvant	Lipopolysaccharide (LPS)	CT26	Complete tumor eradication and prevention of metastasis	24
**PTT**	**IR-7-lipo**	Immunoadjuvant	HA-CpG	CT26	Inhibited tumor growth	25
**PTT**	**PDA, CD**	Immunoadjuvant	Resiquimod (R848)	4T1	Inhibited distant tumor	27
**PTT**	**Gold, Pt**	PD-L1 Blockade	Anti-PD-L1 antibody: LM^D^P	4T1	Suppressed primary and distal tumor growth	57
**PTT**	**IR820**	IDO inhibition	1MT	B16F10, 4T1	Inhibition ratio of 87%	29
**PTT**	**ICG**	Immunoadjuvant	Glycated chitosan	Breast cancer patients	Clinical beneficial response rate was 75% in 1 year	32
**PTT**	**ICG**	Immunoadjuvant	Imiquimod	Melanoma patients	Survival rate reached 70% in 1 year	33
**PTT**	**ICG**	Immunoadjuvant	Imiquimod	Melanoma patients	Eliminated primary tumor and pulmonary metastasis	34
**PTT**	**ICG;** **ICG;** **SWNT;** **None;** **Optical fiber**	Immunoadjuvant	GC	DMBA-4; DMBA-4; EMT6; Panc02-H7; B16	33% tumor-free survival rate; Total resistance to the primary tumors and metastases; Cure rate of 70%; 75% of mice complete tumor regression; The infiltration of TILs increased	44 45 48 49 50
**PTT**	**CuS**	Immunoadjuvant	CpG	EMT6	Destroyed treated tumors and inhibited remote untreated tumors	51
**PTT**	**SWNT**	Immunoadjuvant/CTLA-4 Blockade	GC/ anti-CTLA-4 antibody	4T1	Prolonged the survival time	56
**PTT**	**ICG**	Immunoadjuvant/CTLA-4 Blockade	Imiquimod/ anti-CTLA-4 antibody	4T1	Primary tumors eradication and metastasis prevention	58
**PTT**	**ICG**	Immunoadjuvant	CAR-T cell	WM115	Inhibition the growth of melanoma	59
**PTT**	**ICG**	Immunoadjuvant	Imiquimod	Melanoma patients	No detectable visceral metastasis in 18 months	64
**PTT**	**Focal laser**	Immunoadjuvant	Dinitrophenyl (DNP)	Melanoma patients	Longer overall survival time	66
**PIT**	**IRDye-700DX**	Monoclonal antibody	NZ-1	MPM	Reductions in tumor volume	74
**PIT**	**IRDye-700DX**	Monoclonal antibody	Anti-DLL3 rovalpituzumab	SCLC	Tumor shranked	90
**PIT**	**IRDye-700DX**	Monoclonal antibody	Trastuzumab	Ovarian cancer	Reductions in tumor volume	86
**PIT**	**IRDye-700DX**	Monoclonal antibody	Human IgG1 anti-PSMA mAb	PC3	Prolonged survival time	91
**PIT**	**BPD**	Monoclonal antibody	FDA-approved anti-EGFR mAb	human EOC	Inhibition of early recurrence	82
**PIT**	**IRDye-700DX**	Monoclonal antibody/PD-1 Blockade	Anti-CD44/anti-PD-1 antibody	MC38	Complete rejection of MC38 tumors and distant tumors	92
**PIT**	**IRDye-700DX**	Monoclonal antibody	FDA-approved anti-EGFR mAb	Terminal head and neck cancer patients	Tumor density was decreased in 7/8, with ORR 75% and DCR 100%.	94
**PIT**	**IRDye-700DX**	Monoclonal antibody	FDA-approved anti-EGFR mAb	rHNSCC (in Phase 2)	The ORR was 50% (15/30), with 16.7% (5/30) CR and 86.7% (26/30) DCR	95
**PDT**	**mTHPC/ICG**	Immunoadjuvant	GC	EMT6/ lung adenocarcinoma	Cured 75%/37% of the tumor-bearing mice	47
**PDT**	**Gold**	CTLA-4 Blockade	Anti-CTLA-4 antibody	4T1	Reductions in tumor volume	99
**PDT**	**Levulan**	Immunoadjuvant	Aldara/imiquimod	Basal-cell carcinoma patients	6 cases, 18 cases were completely cured	102
**PDT**	**Methyl aminolevulinate (MAL)**	Immunoadjuvant	Imiquimod cream (IMIQ)	Basal cell carcinoma patient	No sign of the tumor IN two years	103
**PDT**	**Methyl aminolevulinate (MAL)**	Immunoadjuvant	Imiquimod cream (IMIQ)	NMSC patients	MAL-PDT compared to IMIQ (25/44, 56.81% vs. 19/44, 43.19%)	104
**PTT +PDT**	PDA, CAT	Immunoadjuvant	CAT	U14	Complete eradication	60
**PTT +PDT**	Gold, ICG, CAT	Immunoadjuvant	CAT	U14	Complete eradication	100
